# Comparative cytogenetics of ten species of cichlid fishes (Teleostei, Cichlidae) from the Araguaia River system, Brazil, by conventional cytogenetic methods

**DOI:** 10.3897/CompCytogen.v6i2.1739

**Published:** 2012-04-27

**Authors:** G. Targino Valente, C. de Andrade Vitorino, D.C. Cabral-de-Mello, C. Oliveira, I. Lima Souza, C. Martins, P.C. Venere

**Affiliations:** 1 Laboratory of Integrative Genomics, Department of Morphology, Institute of Biosciences, UNESP – Sao Paulo State University, 18618-970, Botucatu, Sao Paulo, Brazil; 2 Laboratory of Ichthyology, Institute of Biological Sciences and Health, Federal University of Mato Grosso, Campus of Araguaia, 78698-000, Pontal do Araguaia, Mato Grosso, Brazil; 3 Department of Biology, Institute of Biosciences, UNESP – Sao Paulo State University, 13506-900, Rio Claro, Sao Paulo, Brazil; 4 Laboratory of Biology and Fish Genetics, Department of Morphology, Institute of Biosciences, UNESP – Sao Paulo State University, 18618-970, Botucatu, Sao Paulo, Brazil

**Keywords:** chromosome evolution, fish chromosomes, genome, Cichlidae

## Abstract

Cichlids represent one of the most species-rich families of fishes and have attracted the attention of evolutionary biologists due to the rapid radiation occurring in some groups and the importance of some species in the world aquaculture. Cytogenetic analysis was conducted in 10 cichlid species from the Araguaia River, Amazon Basin, Brazil. The chromosome number was 2n=48 for all analyzed species except for *Laetacara araguaiae* Ottoni et Costa, 2009 (2n=44). Chromosomal polymorphism was detected only in *Geophagus proximus* (Castelnau, 1855), which exhibits an extra large submetacentric and and a dot-like chromosomes. Moreover, the C-banding revealed a general pericentromeric heterochromatic pattern and some additional blocks for some species. The heterochromatic blocks corresponding to AgNOR bearing regions were observed in all species and also corresponded to CMA_3_ positive blocks, which were observed in terminal regions. Besides the general conserved chromosomal and heterochromatin patterns for South American cichlids, the presence of GC-rich heterochromatin was quite different in the species *Biotodoma cupido* (Heckel, 1840), *Geophagus proximus*, *Retroculus lapidifer* (Castelnau, 1855), *Crenicichla strigata* Günther, 1862 and *Heros efasciatus* Heckel, 1840. The results suggest that independent events of heterochromatin modification occurred during chromosome evolution in the group, regardless of the conservation of macro-chromosomal structure.

## Introduction

The family Cichlidae includes more than 3000 species comprising one of the most species-rich families of vertebrates (Nelson 2006). Cichlids are distributed mainly in Latin America, Africa and Madagascar, with only few species in South India and the Middle East (Genner et al. 2007). Cichlids found in the great eastern lakes of Africa have served as a model system for the study of evolution (Kornfield and Smitth 2000, Kocher 2004, Genner et al. 2007), and several species have received increasing scientific attention because of their great importance to tropical and subtropical aquaculture (Pullin 1991). This family represents a monophyletic group, and the limits and interrelationships of all four subfamilies (Etroplinae, Ptychochrominae, Cichlinae and Pseudocrenilabrinae) are well supported by molecular and morphological data (Smith et al. 2008).

The African and Neotropical cichlids, Pseudocrenilabrinae and Cichlinae, respectively, are both monophyletic and represent sister groups (Smith et al. 2008). There are 51 genera and 406 species recognized in Neotropical cichlids (Kullander 1998, 2003). The most recent proposed phylogeny of the group denotes the tribes Cichlini, Retroculini, Astronotini, Chaetobranchini, Geophagini, Cichlasomatini and Heroini as members of the Cichlinae clade (Smith et al. 2008).

The chromosome numbers of approximately 135 species of cichlids have been determined. Although more than 60% of the species present karyotypes with 2n=48, the diploid number ranges from 2n=32 to 2n=60 (Poletto et al. 2010, for review). African cichlids have a modal diploid number of 2n=44, whereas the modal number for Neotropical cichlids is 2n=48. Even though chromosomal data are known for several cichlid species, the amount of available data is not representative of the high diversity of species in the group. The chromosomal data already published for the Cichlinae clade focus mostly on the description of chromosome morphology and mapping of 45S rDNA (Poletto et al. 2010), and the heterochromatin patterns of only few species are described (Table 1). The aim of this work was to contribute in the study of the heterochromatin patterns of South American cichlids and their possible involvement in karyotypic diversification in the group.

**Table 1.** Synthesis of the cichlid species analyzed with respect to the karyotypic formulae, heterochromatin distribution and CMA_3_ patterns. m/sm, metacentric and submetacentric chromosomes; st/a, subtelocentric and acrocentric chromosomes; mi, microchromosomes; q, the long arm of a chromosome; p, the short arm of a chromosome; PeriC or C, pericentromeric regions; Prox, proximal portion of a chromosome; Term, Terminal portion of a chromosome; Int, interstitial portion of a chromosome; Adj, adjacent region; NOR, nucleolus organizing region; The numbers in the column “Additional blocks” indicate the number of chromosomes with the described pattern; in some cases, the ranking of these chromosomes are indicated in parentheses.

Tribes and species	Origin of animals	2n	Karyotypic formulae	Heterochromatin distribution		CMA_3_ + blocks	References
				General pattern	Additional blocks		
**Cichlini**							
*Cichla piquiti* Kullander et Ferreira, 2006	Das Mortes river, Araguaia basin, MT State, Brazil	48	48st/a	PeriC	NOR; term 2	NOR (term)	This work
*Cichla kelberi* Kullander et Ferreira, 2006	Araguaia river, MT State, Brazil	48	48st/a	C	NOR; int 1 q	absent	Teixeira et al. 2009
*Cichla monoculus* Spix et Agassiz, 1831	Uatumã and Solimões rivers, AM State, Brazil	48	48a	PeriC	NOR; int 1 q	absent	Brinn et al. 2004
*Cichla temensis* Humboldt, 1821	Uatumã and Jaú rivers, AM State, Brazil	48	48a	PeriC	NOR; int 1 q	absent	Brinn et al. 2004
**Retroculini**							
*Retroculus lapidifer lapidifer* (Castelnau, 1855)	Das Mortes river, Araguaia basin, MT State, Brazil	48	48st/a	PeriC	NOR; term 1 q	NOR (term) and PeriC	This work
**Astronotini**							
*Astronotus ocellatus* (Agassiz, 1831)	Tietê river, SP State, Brazil	48	16m/sm + 32st/a	C	NOR	absent	Mazzuchelli and Martins 2009
**Geophagini**							
*Apistogramma trifasciata* (Eigenmann et Kennedy, 1903)	Paraná river, Missiones, Argentina	46	16m/sm + 30st/a	PeriC	absent	absent	Roncati et al. 2007
*Biotodoma cupido* (Heckel, 1840)	Das Mortes river, Araguaia basin, MT State, Brazil	48	4m/sm + 44st/a	PeriC	NOR; some prox blocks	NOR (int)	This work
*Crenicichla britskii* Kullander, 1982	Jupiá river, PR State, Brazil	48	8m/sm + 40st/a	PeriC	NOR; 1 p almost completely heterochromatic (1^st^ pair)	absent	Benzaquem et al. 2008
*Crenicichla strigata* Günther, 1862	Das Mortes river, Araguaia basin, MT State, Brazil	48	6m/sm + 42st/a	PeriC	NOR; some prox blocks	NOR (term) and PeriC	This work
*Crenicichla prope johanna* Heckel, 1840	Negro and Solimões rivers, AM State, Brazil	48	8m/sm + 40st/a	PeriC	NOR; term 1 q (19^th^ pair)	absent	Benzaquem et al. 2008
*Crenicichla cincta* Regan, 1905	Negro and Solimões rivers, AM State, Brazil	48	8m/sm + 40st/a	PeriC	adj NOR	absent	Benzaquem et al. 2008
*Crenicichla iguassuensis* Haseman, 1911	Iguaçu river, PR State, Brazil	48	4m + 4sm + 14st + 26a	PeriC	Some term blocks	NOR	Mizoguchi et al. 2007
*Crenicichla inpa* Ploeg, 1991	Negro and Solimões rives, AM State, Brazil	48	6m/sm + 42st/a	PeriC	adj NOR	absent	Benzaquem et al. 2008
*Crenicichla lepidota* Heckel, 1840	São Gonçalo stream and Polegar lake, RS State, Brazil	48	4m + 4sm + 40st/a	PeriC	term 1 p and 1 q (1^st^ pair); int 1 q (1^st^ pair)	NOR	Perazzo et al. 2010
*Crenicichla lepidota* Heckel, 1840	Porto Rico region, Paraná river basin, PR State, Brazil	48	2m + 4sm + 42st/a	PeriC	int 2 (1^st^ and 5^th^ pairs)	absent	Martins et al. 1995
*Crenicichla lugubris* Heckel, 1840	Negro and Solimões rivers, AM State, Brazil	48	8m/sm + 40st/a	PeriC	NOR; int 1 q (2^nd^ pair)	absent	Benzaquem et al. 2008
*Crenicichla niederleinii* (Holmberg, 1891)	Paraná river, Missiones, Argentina	48	6m/sm + 42st/a	PeriC	absent	absent	Roncati et al. 2007
*Crenicichla reticulata* (Heckel, 1840)	Negro and Solimões river, AM State, Brazil	48	6m/sm + 42st/a	PeriC	adj NOR; int 1 q (10^th^ pair)	absent	Benzaquem et al. 2008
*Crenicichla* sp.1	Iguaçu river, PR State, Brazil	48	4m + 4sm + 14st + 26a	PeriC	Some term blocks	NOR	Mizoguchi et al. 2007
*Crenicichla* sp. 2	Iguaçu river, PR State, Brazil	48	4m + 4sm + 14st + 26a	PeriC	Some term blocks	NOR	Mizoguchi et al. 2007
*Geophagus brasiliensis* (Quoy et Gaimard, 1824)	Socavão and Verde rivers, PR State, Brazil	48	6sm + 42st/a	PeriC/C	absent	NOR	Vicari et al. 2006
*Geophagus brasiliensis* (Quoy et Gaimard, 1824)	Jaguarriaíva river, PR State, Brazil	48	6sm + 42st/a	PeriC/C	Some int blocks	NOR	Vicari et al. 2006
*Geophagus brasiliensis* (Quoy et Gaimard, 1824)	Saco da Alemoa, Gasômero, RS State, Brazil	48	4sm + 44st/a	PeriC	NOR	NOR	Pires et al. 2010
*Geophagus brasiliensis* (Quoy et Gaimard, 1824)	Cambezinho and Três Bocas stream, Tibagi river basin, PR State, Brazil	48	4sm + 44st/a	C	NOR	NOR	Pires et al. 2008
*Geophagus brasiliensis* (Quoy et Gaimard, 1824)	Pirapo river, Paranapanema basin, PR State, Brazil	48	8sm + 40st/a	PeriC	prox 1 p (10^th^ pair)	absent	Martins et al. 1995
*Geophagus proximus* (Castelnau, 1855)	Das Mortes river, Araguaia basin, MT State, Brazil	48	4m/sm + 44st/a	PeriC	NOR; 1 p almost completely heterochromatic	NOR (int)	This work
*Gymnogeophagus balzanii* (Perugia, 1891)	Paraná river, Missiones State, Argentina	48	2m/sm + 46st/a	PeriC	absent	absent	Roncati et al. 2007
*Geophagus gymnogenys* (Hensel, 1870)	Saco da Alemoa, Barra do Ribeiro, Gasômetro, RS State, Brazil	48	4m + 44st/a; 6m + 42st/a	PeriC	NOR	NOR	Pires et al. 2010
*Geophagus labiatus* (Hensel, 1870)	Saco da Alemoa, Forqueta river, RS State, Brazil	48	4m + 4sm + 40st/a	PeriC	absent	NOR	Pires et al. 2010
*Gymnogeophagus* sp.	Paraná river, Missiones, Argentina	48	2m/sm + 46st/a	PeriC	absent	absent	Roncati et al. 2007
*Satanoperca jurupari* (Heckel, 1840)	Das Mortes river, Araguaia basin, MT State, Brazil	48	4m/sm + 44st/a	PeriC	absent	NOR	This work
*Satanoperca pappaterra* (Heckel, 1840)	Porto rico region, Parana river basin, PR State, Brazil	48	6sm + 42st/a	PeriC	absent	absent	Martins et al. 1995
**Cichlasomatini**							
*Aequidens tetramerus* Heckel, 1840	Araguaia river, MT State, Brazil	48	12m/sm + 36st/a	PeriC	absent	NOR	This work
*Australoheros facetus* (Jenyns, 1842)	São Gonçalo stream and Polegar lake, RS State, Brazil	48	22sm + 26st/a	PeriC/C	absent	NOR	Perazzo et al. 2010
*Bujurquina vittata* (Heckel, 1840)	Paraná river, Missiones, Argentina	44	22m/sm + 8st/a + 14 mi	PeriC	NOR; p arm of 5^th^ pair completely heterochromatic	absent	Roncati et al. 2007
*Cichlasoma dimerus* (Heckel, 1840)	Paraná river, Missiones, Argentina	48	8m/sm + 40st/a	PeriC	absent	absent	Roncati et al. 2007
*Cichlasoma facetum* (Jenyns, 1842)	Tarumã lake, PR State, Brazil	48	10sm + 38 st/a	PeriC/C	absent	NOR	Vicari et al. 2006
*Cichlasoma paranaense* Kullander, 1983	Porto rico region, Parana river basin, PR State, Brazil	48	20sm + 28 st/a	PeriC	prox 2 p (2^nd^ and 9^th^ pairs)	absent	Martins et al. 1995
*Laetacara araguaiae* Ottoni et Costa, 2009	Araguaia river, MT State, Brazil	44	4m/sm + 40st/a	PeriC	absent	NOR	This work
*Laetacara* prope *dorsigera* (Heckel, 1840)	Paraná river, PR State, Brazil	43	5m + 38a	C	NOR	absent	Martins-Santos et al. 2005
		44	4m + 40a				
		45	3m + 42a				
		46	2m + 44a				
**Heroini**							
*Heros efasciatus* Heckel, 1840	Araguaia river, MT State, Brazil	48	8m/sm + 40st/a	PeriC	absent	NOR (term) and int 1 p	This work
*Mesonauta festivus* (Heckel, 1840)	Das Mortes river, Araguaia basin, MT State, Brazil	48	14m/sm + 34st/a	PeriC	NOR; term 2 q	NOR (term)	This work
*Pterophyllum scalare* (Schultze, 1823)	Jari river, PA State, Brazil	48	12m/sm + 36st/a	PeriC/C	1 p almost completely heterochromatic (1^st^ pair)	NOR, some centromeres	Nascimento et al. 2006
*Symphysodon aequifasciatus* Pellegrin, 1904	Bauana lake, Tefé river, AM State, Brazil	60	8m/sm + 8st/a +4mi; 50m/sm + 6st/a +4mi	PeriC	Some prox blocks; int 1 q (1^st^ pair)	absent	Mesquita et al. 2008
*Symphysodon discus* Heckel, 1840	Boi-boi stream, Negro river, AM State, Brazil	60	50m/sm + 10st/a; 54m/sm + 6st/a	PeriC	Some prox blocks	absent	Mesquita et al. 2008
*Symphysodon haraldi* Schultz, 1960	Manacapuru river, AM State, Brazil	60	52m/sm + 4st/a +4mi	PeriC	Some prox blocks	absent	Mesquita et al. 2008

## Material and methods

### Specimens and chromosome preparation

It was analyzed 10 South American cichlid species of the subfamily Cichlinae: *Cichla piquiti* Kullander et Ferreira, 2006 (4 individuals: sex not identified), *Retroculus lapidifer* (Castelnau, 1855) (6 individuals: 3 ♀ and 1 ♂, and 2 sex not identified), *Biotodoma cupido* (Heckel, 1840) (5 individuals: 2 ♀, and 3 ♂), *Crenicichla strigata* Günther, 1862 (12 individuals: 5 ♀, 5 ♂, and 2 sex not identified), *Geophagus proximus* (Castelnau, 1855) (9 individuals: 4 ♀, 2 ♂, and 3 sex not identified), *Satanoperca jurupari* (Heckel, 1840) (15 individuals: 7 ♀, 5 ♂, and 3 sex not identified), *Aequidens tetramerus* Heckel, 1840 (44 individuals: 21 ♀, 14 ♂, and 9 sex not identified), *Laetacara araguaiae* Ottoni et Costa, 2009 (5 individuals: 1 ♀, 1 ♂, and 3 sex not identified), *Heros efasciatus* Heckel, 1840 (5 individuals: 5 females) and *Mesonauta festivus* (Heckel, 1840) (5 individuals: 2 ♀, 1 ♂, and 2 sex not identified), which belong to the tribes Cichlini, Retroculini, Geophagini, Cichlasomatini and Heroini (Table 1). All individuals analyzed were not juveniles. Wild specimens were collected in several rivers that are part of the Araguaia River system, which is situated in the quadrant bounded by the coordinates 52°24'00"W, 15°30'S (DMS) and 52°05'00"W, 15°58'S (DMS) in the region of Barra do Garças, Mato Grosso State, Brazil. The sampling of wild animals was performed in accordance with Brazilian laws for environmental protection (wild collection permit, SISBIO/15729–1). The animals were maintained for 24 hours in an aired aquarium at a temperature ranging from 25°C to 28°C before collecting tissue samples. The fish were euthanized with a lethal dose of benzocaine followed by spinal section (Protocol 01204 – Committee of Ethical in Animal Experimentation – UNESP – São Paulo State University, Brazil) before removal of the kidneys for chromosome preparation.

Mitotic chromosome preparations were obtained from kidney cells according to Bertollo et al. (1978). The animals were treated with a 0.0125% solution of colchicine, which was injected at a volume of 1mL/100g of body weight at approximately 45–60 min before euthanasia and chromosome preparation. The kidney tissues were dissected, and the cells were dissociated in a hypotonic solution of KCl 0.075 M with a syringe and remained in the solution for 25 min. The cells were fixed in 3:1 methanol-acetic acid solution and used to prepare slides that were stained with 5% Giemsa solution in phosphate buffer at pH 7 for 10 min.

### Differential chromosome staining and banding

The chromosome structure was analyzed through silver nitrate staining, Chromomycin A_3_ (CMA_3_) staining and C-banding.

To detect nucleolus organizer regions (NORs), the silver staining of the chromosomes was performed according to Howell and Black (1980). The slides were stained with 2% Giemsa for 10 to 15 sec, washed in water and air-dried for later microscopic analysis.

The constitutive heterochromatin was detected using saline solution according to Sumner (1972) with the following adjustments. The slides were initially treated with 0.2 N HCl at 42°C for 5 min, washed in water and rapidly air-dried. The slides were then immersed in 5% barium hydroxide solution that was freshly prepared and filtered at 42°C for 30 sec to 1 min. The treatment was stopped by submerging the slides in 0.2 N HCl and washing them extensively in running water. The slides were immersed in saline solution (2xSSC) at 60°C for 45 min. After completing this step, the slides were air-dried and stained with 5% Giemsa in phosphate buffer at pH 6.8–7.0. Alternatively, the slides were stained with propidium iodide, which also provides excellent results.

The CMA_3_ staining was conducted according to the method by Schweizer (1976) with minor adjustments. This was done by immersing the slides in 0.2% MgCL_2_ in McIlvaine buffer, pH 7.0, at 25°C for 10 min. The slides were withdrawn, agitated briefly to remove excess solution, mounted with 150 µL of 0.05% CMA_3_ in McIlvane buffer under coverslips and then stored in dark boxes for 15 min at 25°C. After this step, the coverslips were removed by washing the slides in McIlvaine buffer. The slides were incubated in a solution of freshly prepared of 0.012% Methyl-green/Hepes for 15 min, rinsed in a solution of Hepes 0.13%/NaCl 0.87% and air-dried. Finally, the slides were mounted with 45–90 µl of glycerol 97.4%/propyl gallate 2.5%. Prior to analysis, the slides were stored in the dark at 4°C for at least one week before analysis by fluorescence microscopy.

### Chromosome analysis

The chromosome spreads were analyzed using an Olympus BX 61 microscope, and the images were captured with the Olympus DP71 digital camera with the software Image-Pro MC 6.0. There were analyzed 30 metaphase spreads for all cytogenetic procedures performed for each animal sample. Karyotypes were arranged in the order of decreasing chromosome size, and the chromosomes were classified as either meta/submetacentrics (m/sm) or subtelo/acrocentrics (st/a).

## Results

**Figure 1.**
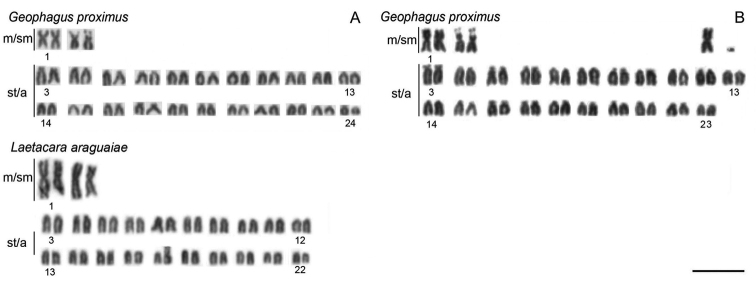
Representative karyotypes of *Geophagus proximus* and *Laetacara araguaiae* species. For *Geophagus proximus*,two karyotypes are presented, a normal (**A**) and a polymorphic karyotype, showing in the upper right corner one extra large metacentric and one dot-like chromosome (**B**). Bar = 10 µm.

All of the species analyzed have 2n=48 except *Laetacara araguaiae*, which showed a diploid number of 2n=44 and the karyotype formula of 4m/sm + 40st/a. Moreover, chromosomal polymorphism was found in *Geophagus proximus*, which presented two karyotype formulae, 4m/sm + 44st/a or 5m/sm + 42st/a + 1 dot-like chromosome (Fig. 1, Table 1).

**Figure 2.**
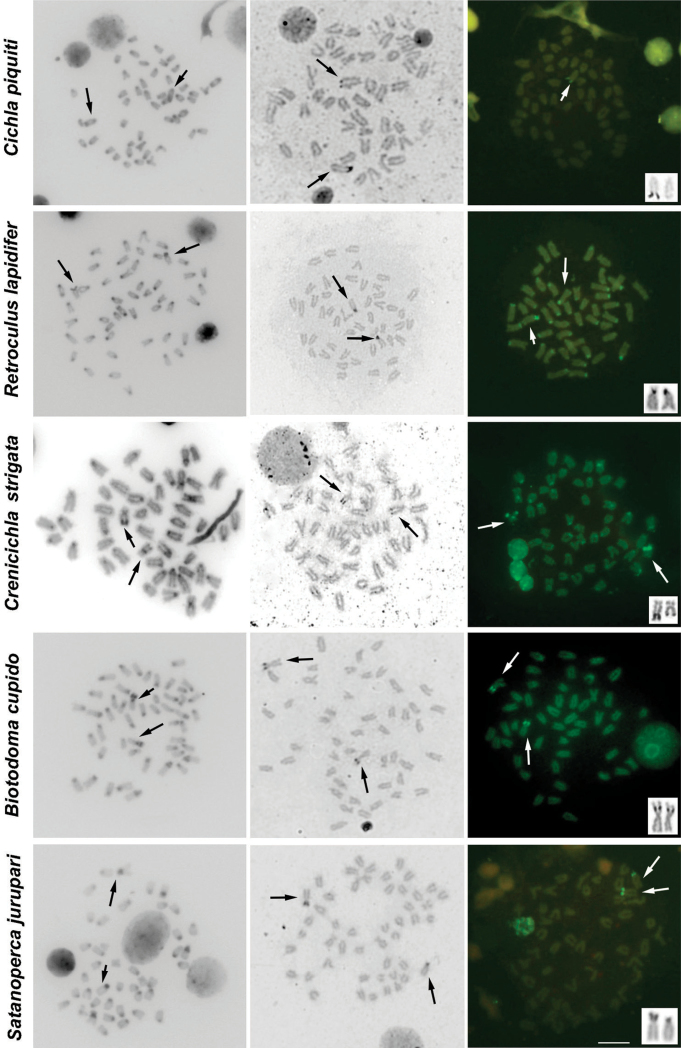
Metaphases of several cichlid species under different chromosome treatments. The species are indicated on the left. The first, second and third columns show C-banded, AgNOR- and CMA_3_- stained metaphases, respectively. The third column shows chromosomes bearing AgNORs in the box. The arrows indicate the NOR-bearing chromosomes. Bar = 10 μm.

**Figure 3.**
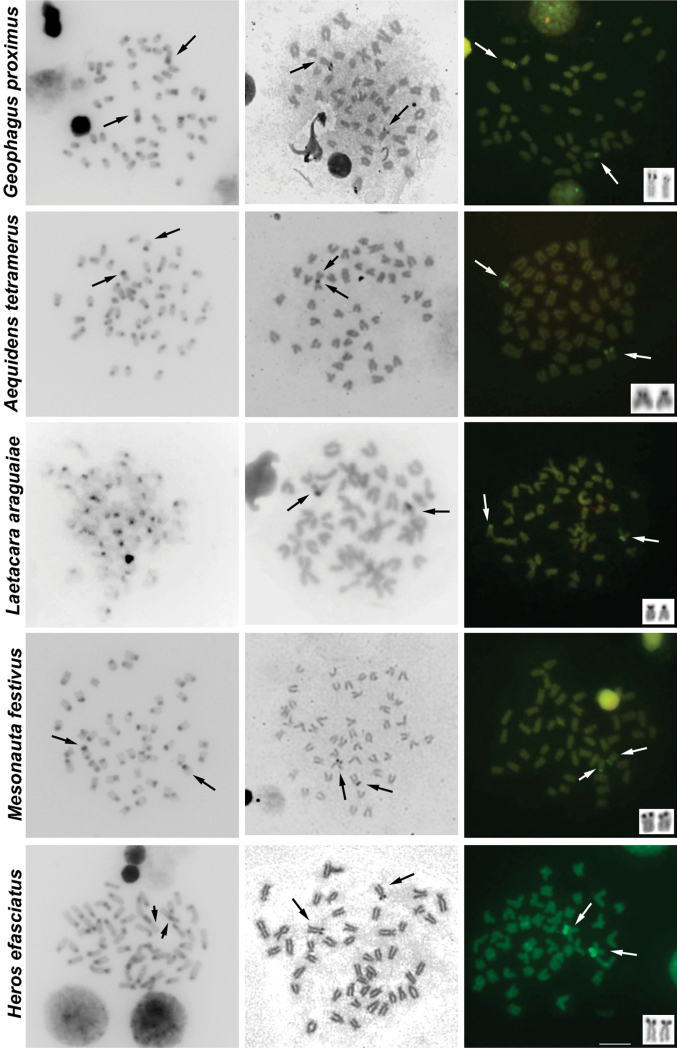
Metaphases of several cichlid species under different chromosome treatments. The species are indicated on the left. The first, second and third columns show C-banded, AgNOR- and CMA_3_- stained metaphases, respectively. The third column shows chromosomes bearing AgNORs in the box. The arrows indicate the NOR-bearing chromosomes. For some metaphases (without arrows) it was not possible to identify the NOR-carrying chromosomes. Bar = 10 µm.

The results of C-banding revealed the heterochromatin generally restricted to pericentromeric regions. Additional blocks of heterochromatin were noticed in *Cichla piquiti*, *Retroculus lapidifer*, *Biotodoma cupido*, *Crenicichla strigata*, *Geophagus proximus* and *Mesonauta festivus* (Figs 2, 3, Table 1).

Characteristic heterochromatic blocks corresponding to AgNOR bearing regions (two blocks, one in each homologue) were observed in all species, and these blocks were consistent with CMA_3_ positive (CMA_3_^+^) blocks (Figs 2, 3, Table 1). These AgNOR/CMA_3_^+^ blocks were present in terminal regions; however, positional variation was observed in *Biotodoma cupido* (Fig. 2) and *Geophagus proximus* (Fig. 3), which the blocks are present in interstitial regions. Moreover, *Retroculus lapidifer* (Fig. 2) and *Crenicichla strigata* (Fig. 2) displayed CMA_3_^+^ blocks in pericentromeric regions of almost all chromosomes, and *Heros efasciatus* (Fig. 3) displayed a positive interstitial signal in one chromosome pair. Size variation was also observed in AgNOR/CMA_3_^+^ blocks between homologous chromosomes in *Cichla piquiti* (Fig. 2), *Crenicichla strigata* (Fig. 2) and *Satanoperca jurupari* (Fig. 2). Other chromosomal areas were CMA_3_ neutral in all of the species analyzed (Figs 2, 3).

## Discussion

The diploid number reported for the species in this study, in general are in agreement with the conserved 2n=48 chromosomes commonly found in South American cichlids and in contrast with the presence of 2n=44 chromosomes in African cichlids. All species, except *Laetacara araguaiae*, had their diploid number already described (Poletto et al. 2010). Moreover, some cichlid species display the occurrence of specific chromosomal rearrangements, such as pericentric inversions, translocations and fission or fusion rearrangements, that occurred during their evolutionary history and deviate their karyotypic formulae from common pattern observed for cichlids (revised by Feldberg et al. 2003, Mesquita et al. 2008, Poletto et al. 2010).

Chromosomal variability was observed in derived lineages, such as the Geophagini and the Cichlasomatini tribes (Feldberg et al. 2003, Poletto et al. 2010). Thus, the diploid number variation observed here in *Laetacara araguaiae* and the polymorphism observed in *Geophagus proximus*, which belong to Cichlasomatini and Geophagini tribes, respectively, could reflect the higher chromosomal variation found in these tribes. In fact, another species of *Laetacara* Kullander, 1986, *Laetacara prope dorsigera* (Heckel, 1840),generally displayed 2n=44 chromosomes with an intraspecific variation in the diploid number that ranges from 2n=43 to 2n=46, which are thought to have originated from centric chromosomal fusions (Martins-Santos et al. 2005). In *Geophagus proximus*, the polymorphism is a consequence of a Robertsonian translocation between two st/a chromosomes that results in a large metacentric chromosome and a dot-like element. However, it is inconclusive if this rearrangement occurred between homologous or non-homologous chromosomes due to the great similarities among the st/a chromosomes in *Geophagus proximus*.

Chromosomal rearrangements such the ones reported here could lead to the karyotypic diversification of the species. In fact, chromosomal rearrangements have contributed to karyotypic evolution in a range of fishes, including the cichlids *Symphysodon* (Heckel, 1840) (Mesquita et al. 2008, Gross et al. 2009a), salmonids (Allendorf and Thorgaard 1984) and *Gobius fallax* Sarato, 1889(Thode et al. 1988), among others. Moreover chromosomal rearrangements may result in intraspecific variation as broadly reported in some fish species: in the origin of neo-Y sex chromosomes (Uyeno and Miller 1971, 1972, Bertollo et al. 1983, 1997, Almeida-Toledo et al. 1984, 1988, 2000, Silva and Margarido 2005), in karyotypic diversification of species complex of *Gymnotus carapo* Linnaeus, 1758 (Milhomem et al. 2008), in *Hoplias malabaricus* (Bloch, 1794) (Bertollo et al. 1997) and in *Erythrinus erythrinus* (Bloch et Schneider, 1801) (Bertollo et al. 2004).

Although the cichlid cytogenetics suggests that the ancestral karyotype (2n=48 st/a) could have undergone major changes (pericentric inversions, fusions, fissions and chromosomal translocations) in the macro-structure of the South American species (Feldberg et al. 2003, Poletto et al. 2010), these studies show that this family of fish has a relatively conserved diploid number. Despite of the absence of conclusive data about chromosomal rearrangements rate that occurs in cichlids, it could be suggested that this group has an intermediate level of chromosomal stability compared to birds and mammals, which are more stable and variable, respectively. It is predicted that chromosomal rearrangements can be one of the evolutionary forces that affect the reproductive isolation and speciation processes (Noor et al. 2001, Rieseberg 2001), which create higher levels of species diversity. However, birds and cichlids display greater species richness than what is observed in mammals; this is contrary to the more stable karyotypes of birds and cichlids. Therefore chromosomal rearrangements may be not the most decisive evolutionary process in the cichlids speciation.

C-banding analyses in this study revealed that the conserved pattern of heterochromatin distribution was mostly restricted to the pericentromeric regions of cichlid chromosomes, which has been commonly reported in American and African representatives but with variations in both groups (Kornfield et al. 1979, Majumdar and McAndrew 1986, Feldberg et al. 2003, and others reported in Table 1). Additional heterochromatic blocks were present in almost all species analyzed, and exceptions were observed in *Satanoperca jurupari* (Geophagini), *Aequidens tetramerus* (Cichlasomatini), *Laetacara araguaiae* (Cichlasomatini) and *Heros efasciatus* (Heroini). For all species, one of these blocks was related to AgNOR regions, which seems to be a common feature in cichlids and other fish (Pendás et al. 1993, Artoni et al. 2008, Souza et al. 2008, Venere et al. 2008, among others cited in Table 1).

Concerning the singular heterochromatic blocks reported here, *Cichla piquiti*, *Crenicichla strigata* and *Geophagus proximus* show variability in the positions, extensions and number of these blocks compared to the other species in each genus. Moreover, the divergent patterns are observed in *Crenicichla* Heckel, 1840 and *Geophagus* Heckel, 1840. This variability can be also observed in the *Laetacara* genus; in this case, *Laetacara araguaiae* does not have any additional heterochromatic blocks, whereas *Laetacara prope dorsigera* has heterochromatic NORs as additional blocks (Martins-Santos et al. 2005). Moreover, both of the *Satanoperca* Günther, 1862 species analyzed do not have any additional heterochromatic blocks. Comparisons within every genera *Retroculus* Eigenmann et Bray, 1894, *Biotodoma* Eigenmann et Kennedy, 1903, *Aequidens* Eigenmann et Bray, 1894, *Heros* Heckel, 1840 and *Mesonauta* Günther, 1862 are not possible because this is the first C-banding analysis for these genera. Heterochromatic variations can be observed when comparing the additional heterochromatic blocks patterns within the tribes Geophagini, Cichlasomatini and Heroinitribes. This analysis could support the current idea that these groups display some of the highest chromosomal variability for the Cichlidae family (Feldberg et al. 2003, Poletto et al. 2010). However, they are the most studied group concerning heterochromatin analysis, and it is not clear if this variability reflects higher chromosomal variability or a sampling effort (for all comparisons see Table 1).

The fluorochrome CMA_3_ showed the presence of GC-rich blocks coinciding with AgNOR sites in all species, which is a common trait in cichlids. The variation in the extension of these blocks also matches the size variation in the AgNOR sites in some species. Additional CMA_3_^+^ blocks are uncommon patterns in cichlids species, but they have been reported here for some species. In addition, this trait has only been previously reported in the Heroini species *Pterophyllum scalare* (Schultze, 1823) (Nascimento et al. 2006). The general pattern of base-pair richness of the heterochromatin indicates some level of compartmentalization of this genomic content at both intragenomic and intraspecific levels. Finally, based on the present and previously reported data, it seems possible that there is a relationship between CMA_3_^+^ blocks and AgNOR regions in cichlid species. Furthermore, the variation may be an exception in this group of fish and could suggest that the sequences presented in these regions may possess some dynamism in cichlids genomes.

With respect to AgNOR, length variation between homologous chromosomes could be explained by the duplication or deletion of 45S rDNA repeat units. All AgNOR sites in the species analyzed here are heterochromatic as aforementioned. The length variation detected and extensively observed in other organisms may be caused by the presence of repetitive sequences, errors during the replication process, unequal crossing-over (Ashley and Ward 1993, Pendás et al. 1993, Boron et al. 2006, Gross et al. 2010) and likely non-reciprocal translocation between these regions (revised in Wasko and Galetti 2000).

## Conclusion

The heterochromatin, CMA_3_^+^ blocks and AgNOR regions are classic cases of enriched repetitive elements regions, such as satellite DNA, transposable elements, and rDNA. Among cichlids, it has been reported that the pericentromeric regions, which are commonly evidenced by C-banding, are repositories for a great amount of repetitive elements, such as transposable elements (Gross et al. 2009b, Mazzuchelli and Martins 2009, Teixeira et al. 2009, Valente et al. 2011). Repetitive sequences are highly dynamic in genome evolution; for example, pericentromeric DNA are rapidly evolving regions in eukaryotic genomes (Haaf and Willard 1997, Csink and Henikoff 1998, Murphy and Karpen 1998) due to the accumulation of repetitive sequences by recombination suppression (Topp and Dawe 2006, Grewal and Jia 2007). In fact, the results reported here and in previous work do not show any phylogenetic relationships in terms of constitutive heterochromatin, NOR and CMA_3_^+^ blocks; therefore, the actual number, position and length variation of sites are not related to any homology. All of the variation observed in these regions may be related to the intrinsic dynamism of repeated sequences and independent heterochromatin modifications that do not follow the diversification of taxa.

## Appendix 1

Synthesis of the cichlid species analyzed with respect to the karyotypic formulae, heterochromatin distribution and CMA3 patterns. Table with synthesis of karyotypic traits of cichlids. File format: Microsoft Excel Spreadsheet (xsl).
